# Healthier Dietary Patterns Are Associated with Better Sleep Quality among Shanghai Suburban Adults: A Cross-Sectional Study

**DOI:** 10.3390/nu16081165

**Published:** 2024-04-13

**Authors:** Li Huang, Yonggen Jiang, Zhongxing Sun, Yiling Wu, Chunxia Yao, Lihua Yang, Minhua Tang, Wei Wang, Nian Lei, Gengsheng He, Bo Chen, Yue Huang, Genming Zhao

**Affiliations:** 1 Songjiang District Center for Disease Control and Prevention, Shanghai 201620, China; lilyh_fudan@163.com (L.H.); sjjkzx1106@126.com (Y.J.); sun983357@163.com (Z.S.); areis2119@163.com (Y.W.); ysan_0720@163.com (C.Y.); yahe_rise@163.com (L.Y.); 20211020086@fudan.edu.cn (M.T.); 2 Xinqiao Community Health Service Center in Songjiang District, Shanghai 201612, China; korl_wang008@163.com (W.W.); 18964549105@163.com (N.L.); 3 Department of Nutrition and Food Hygiene, Key Laboratory of Public Health Safety of Ministry of Education, School of Public Health, Fudan University, Shanghai 200032, China; gshe@shmu.edu.cn (G.H.); chenb@fudan.edu.cn (B.C.); 4 Department of Food Science and Nutrition, Shanghai Business School, Shanghai 200235, China; 5 Department of Epidemiology, Key Laboratory of Public Health Safety of Ministry of Education, School of Public Health, Fudan University, Shanghai 200032, China

**Keywords:** dietary pattern, sleep quality, CHEI, MD, DASH, dairy products, fruits, beverage, Chinese

## Abstract

Background: More is to be explored between dietary patterns and sleep quality in the Chinese adult population. Methods: A cross-sectional study including 7987 Shanghai suburban adults aged 20–74 years was conducted. Dietary information was obtained using a validated food frequency questionnaire. Adherence to a priori dietary patterns, such as the Chinese Healthy Eating Index (CHEI), Dietary Approaches to Stop Hypertension (DASH) diet and Mediterranean diet (MD), was assessed. Sleep quality was assessed from self-reported responses to the Pittsburgh Sleep Quality Index (PSQI) questionnaire. Logistic regression models adjusting for confounders were employed to examine the associations. Results: The overall prevalence of poor sleep (PSQI score ≥ 5) was 28.46%. Factor analysis demonstrated four a posteriori dietary patterns. Participants with a higher CHEI (OR_Q4 vs. Q1_: 0.81, 95% CI: 0.70–0.95), DASH (OR_Q4 vs. Q1_: 0.70, 95% CI: 0.60–0.82) or MD (OR_Q4 vs. Q1_: 0.75, 95% CI: 0.64–0.87) had a lower poor sleep prevalence, while participants with a higher “Beverages” score had a higher poor sleep prevalence (OR_Q4 vs. Q1_: 1.18, 95% CI: 1.02–1.27). Conclusions: In Shanghai suburban adults, healthier dietary patterns and lower consumption of beverages were associated with better sleep quality.

## 1. Introduction

Good sleep is critical for both mental and physical well-being, as well as a high quality of life, which plays a crucial role in maintaining human homeostasis and promoting health [1]. But a lot of things about modern life, like staying up late working, engaging in more activities at night, and using screens for electronic media more often, can cause sleep issues [2,3,4]. Numerous studies have shown a constant link between poor sleep quality and multimorbidity, such as mental dysfunctions, cardiovascular disease, diabetes and obesity [5]. In recent years, inadequate sleep—in terms of both quality and quantity—has raised more and more worries about public health worldwide [6].

The dynamics of sleep and wake are strongly linked to the circadian rhythm, which oscillates in cycles of 24 h [7]. The body’s biological clock’s internal genetic components, or clock genes, as well as external influences from the environment and diet, together regulate the circadian rhythm [8]. Given the correlation between sleep-related behaviors and unfavorable health consequences, it is imperative to examine and ascertain the putative dietary factors that influence sleep quality. It is thought that diet has a significant impact on how effectively sleep is regulated [9]. Poorer sleep was linked to higher dietary energy intake or poorer diet quality, according to a systematic review that examined the potential bidirectional association between sleep and dietary intake. Conversely, the results regarding how diet might affect sleep were less consistent when taking into account energy and macronutrient intake, or micronutrient and small metabolite intake [10]. Additionally, a systematic summary of the evidence investigated the relation between diet-related variables as the exposure and sleep quality features as the outcome, which summarized that eating a healthy diet was associated with improved sleep quality, while consuming a higher intake of processed and sugar-rich foods was associated with worse sleep features [11]. However, a different systematic evaluation of nutritional interventions aimed at promoting sleep behavior revealed conflicting results regarding the efficacy of nutrition as a sleep-enhancing treatment [12]. These inconclusive results suggested the complicated correlation between dietary intake and sleep quality, which deserves further exploration. Furthermore, it is imperative to investigate the relationship between sleep quality and diet, since sleep quality cannot be autonomously improved easily, while diet quality can.

Nutrient or food category analysis used to be a standard procedure in the field of nutritional epidemiology. However, foods and nutrients are ingested in combination rather than separately within dietary patterns. This discipline has undergone a revolution with the advent of dietary patterns and their analysis, which has allowed for the complex interactions between nutrients and foods as well as the consideration of the synergistic effects of foods [13]. There are two ways to derive dietary patterns: an a posteriori approach that uses statistical exploratory methods to identify dietary patterns based on an individual’s dietary intake and an a priori approach that scores an individual’s adherence to the recommended dietary guidelines or predefined dietary pattern [14]. Dietary patterns, as opposed to specific foods or nutrients, are predicted to have a more profound and varied impact on biological and behavioral processes, making them more predictive of overall health status and disease risk [15], which are then most often investigated with respect to their relation to sleep.

The relationship between sleep and diet is difficult to determine in terms of cause and effect, and these two behaviors may well influence one another. However, considering that diet quality can be actively enhanced more easily, many studies tended to use dietary factors as an independent variable. Moreover, altering the dietary pattern overall is considered a more sustainable lifestyle option than merely adding to or subtracting from a certain food or nutrient intake [16]. There is emerging information surrounding the link between diet patterns and sleep in different populations. Studies have demonstrated that following a Mediterranean diet (MD) is linked to improved sleep quality and a decreased risk of insomnia in European adults [17,18], and that an MD pattern is predictive of improved sleep quality in US women [19]. A study found that persons in the US who followed the Dietary Approaches to Stop Hypertension (DASH) diet had longer and better-quality sleep [20]. Higher diet quality, as shown by the dietary indices, was substantially correlated with better sleep quality [21,22,23,24]. Moreover, other a posteriori dietary patterns associated with good sleep quality have also been reported [25,26,27]. There is currently minimal research on the connection between Chinese people’s sleep quality and their a priori MD or DASH food patterns. Given the different socioeconomic and cultural characteristics of Chinese populations, it is uncertain if the linked findings from earlier research would apply to them. Recently, several studies using an a posteriori approach reported diet quality and specific food consumption had been linked to sleep quality in Chinese populations [28,29,30]. These findings were intriguing, on the one hand, as the effect of dietary patterns on sleep problems had been reported, and on the other hand, as the dietary patterns related to sleep quality varied considerably across different study populations, and more studies are necessary to confirm the relationship between diet and sleep. Therefore, the purpose of this study was to explore the cross-sectional association between both a priori and a posteriori dietary patterns and sleep quality among adults of a suburban community in Shanghai. The results would lay a foundation for future research to identify the key dietary factors affecting sleep quality in this population, which may provide a theoretical basis for improving sleep quality through nutritional interventions and dietary guidance for community residents in the future.

## 2. Materials and Methods

### 2.1. Study Design and Population

Participants were drawn from the Shanghai Suburban Adult Cohort and Biobank (SSACB) survey’s Xinqiao community. The purpose of the SSACB survey was to investigate genetic, behavioral, and environmental risk factors for noncommunicable chronic diseases (NCDs) in individuals (20–74 years old) residing in a rapidly urbanizing suburban area of Shanghai, which has been extensively documented previously [31]. The target population, which consisted of native Chinese citizens who had either registered as a Shanghai household or had been in Shanghai for at least five years, was chosen at random through a multi-stage, stratified, clustered sampling process. Between April 2016 and October 2017, the cohort examined 44,887 participants in-person and performed clinical examinations. The Xinqiao Communities sample was one of the seven sample communities that responded to a baseline survey that included 9257 respondents. This cross-sectional study included participants who completed a detailed sleep questionnaire and a food consumption assessment. Following the exclusion of 1270 individuals with missing or incomplete information from the FFQ survey, sleep questionnaire, physical examination, or demographics, as well as those with unreasonable energy intake (male: <800 kcal or >4000 kcal, female: <500 kcal or >3500 kcal) [32], there were 7987 samples in the analysis, and 86.28% of respondents responded. The Fudan University Institutional Review Board gave its approval for this study, which was carried out in accordance with the Declaration of Helsinki (IRB#2016-04-0586). Every participant provided written informed consent after receiving a thorough description of the processes.

### 2.2. Questionnaire and Anthropometric Measurements

During the baseline survey, a standardized questionnaire interview was used to gather data on sociodemographic traits, lifestyle, past health, and food consumption. Investigations were conducted into the sociodemographic data, including age, sex, education, and marital status. Lifestyle factors included the frequency of physical activity, consumption of alcohol and tobacco products, and sedentary habits. Self-reported histories of diabetes, hypertension, hyperlipidemia, chronic bronchitis, heart disease, stroke, asthma, renal disease, Parkinson’s, Alzheimer’s, depression, and schizophrenia were among the health status factors. Each subject was asked to stand without shoes and wear light clothing so that a trained health worker could measure their body weight to the nearest 0.1 kg. An electronic height and weight scale was used to measure the participant’s height to the nearest 0.1 cm while they were standing. The formula for calculating the body mass index (BMI) was body weight in kg/(height in m)^2^. A validated, semi-quantitative food frequency questionnaire (FFQ) was used to collect data on food consumption; the FFQ’s validity and reliability had already been established [33,34].

### 2.3. Sleep Quality

Using self-reported answers to the Pittsburgh Sleep Quality Index (PSQI) questionnaire [35], sleep quality was evaluated at baseline. Research has demonstrated the great reliability and validity of the PSQI as a screening tool for sleep disorder in both clinical and non-clinical samples [36]. The PSQI was utilized to evaluate the preceding month’s sleep quality. The 19 elements that make up the composite score comprise 7 subcomponent scores: subjective sleep quality, sleep latency, duration, habitual sleep efficiency, sleep disturbances, sleep medication, and daytime functioning. Higher scores indicate lower overall sleep quality. Each subcomponent was assigned a value between 0 and 3, with an overall total score of 0 to 21. Subjects with a PSQI score of ≥5 were classified as poor sleepers, whereas those with a score of <5 were classified as good sleepers [24].

### 2.4. A Priori Dietary Pattern Scores

The 3 diet quality scores—the Mediterranean diet (MD), the Dietary Approaches to Stop Hypertension (DASH) diet, and the Chinese Healthy Eating Index (CHEI)—were computed for adherence. Details of the 3 scores have been provided previously [33,34]. Higher scores indicate better adherence to the dietary pattern. The three scores range from 0 to 9 (MD), 8 to 40 (DASH) and 0 to 100 (CHEI) (Supplementary Materials, Table S1).

### 2.5. A Posteriori Dietary Pattern Scores

The dietary patterns were identified using factor analysis. Initially, based on similarity in food type and nutrient composition, individual food items from the FFQ were combined into 16 food categories (Supplementary Materials, Table S2). The 16 food groups were used in a varimax rotation factor analysis. A scree plot, the interpretability, and eigenvalues greater than 1.0 were used to retain factors. The dietary pattern was given a name based on each factor’s increased factor load (FL > 0.4). Subsequently, the factor loadings of the food items were taken into account to determine the factor scores for each pattern for every participant. A higher score indicated more adherence to the derived pattern. The current investigation identified four dietary patterns, namely “Protein-rich and vegetables”, “Dairy and fruits”, “Snacks, salted food and nuts” and “Beverages” dietary pattern, which explained 16.776%, 8.769%, 7.375% and 7.000% of the variation, respectively (Table 1).

### 2.6. Considered Covariates

We collected data on sociodemographic factors (age, sex, education level, marital status and BMI), lifestyle factors (tobacco smoking, alcohol drinking, physical exercise and being sedentary), and physical health-related factors (hypertension, hyperlipidemia, diabetes, coronary heart disease, stroke, urarthritis, asthma, kidney disease, cancer, schizophrenia, Parkinson’s disease, Alzheimer’s disease, depressive disorder).

Based on the residents’ height and weight, the body mass index (BMI, kg/m^2^) was computed using the formula = weight (kg)/height^2^ (m^2^). Based on the reference standard of the Chinese body mass index, the residents’ BMI was divided into four categories: underweight (<18.5), normal (18.5–23.9), overweight (24–27.9), and obese (>28). Smoking was defined as consuming one cigarette or more every day for a minimum of six months. The definition of alcohol consumption was three drinks a week or more for a minimum of six months. The information about types and durations of participants’ weekly physical activities was collected in detail. Exercise was defined as performing physical activity (lasting at least 10 min) per week [31]. The existing hospital diagnostic that was in place served as the basis for the diagnosis of non-communicable chronic diseases.

### 2.7. Statistical Analysis

The a posteriori dietary patterns were identified by factor analysis. To determine the demographic features of the sample, descriptive statistics were used. The results were displayed as means ± SD, or as numbers and percentages for categorical data. Using descriptive analysis, the normality of the data was examined. The study employed Student’s independent *t* test, one-way ANOVA, and chi-square test to investigate participant group differences. Participants were divided into four groups (Q1–Q4) based on the quartiles of the three a priori dietary pattern scores and four a posteriori dietary pattern scores. These groups represented varying levels of adherence, with Q1 having the lowest adherence and Q4 having the highest adherence. Chi-square tests were conducted to examine the distribution differences between good and poor sleep quality status among these groups. Logistic regression models were used to assess the associations between each eating pattern score group and poor sleep quality. These models were further adjusted for factors that may impact sleep quality. The odds ratios (ORs) along with their corresponding 95% confidence intervals (CIs), were calculated using the first quartile group (Q1, representing the lowest diet quality) as the reference. A two-sided *p*-value of less than 0.05 was deemed statistically significant. To conduct the statistical analyses, SPSS v20.0 (SPSS Inc., Chicago, IL, USA) was used.

## 3. Results

### 3.1. Characteristics of Study Participants

In this study, 7987 inhabitants between the ages of 20 and 74 were included; their mean age was 53.19 ± 13.47 years, their mean BMI was 23.68 ± 4.91 kg/m^2^, and their mean daily calorie consumption was 1408.39 ± 579.45 kcal. There were 3117 males (39.03%) and 4870 (60.97%) females. The majority of participants (79.84%) were between the ages of 40 and 74; 92.45% were married; and 65.87% had completed secondary school or higher. Drinking was prevalent in 9.85% of cases and smoking in 20.90% of cases, respectively. Just 23.50% of the individuals were sedentary for more than six hours a day, whereas 71.15% of them engaged in physical activity for at least thirty minutes per week. Many of them suffered from a variety of chronic illnesses (Table 2).

### 3.2. Pittsburgh Sleep Quality Index Scores for Study Participants

**Figure 1:**
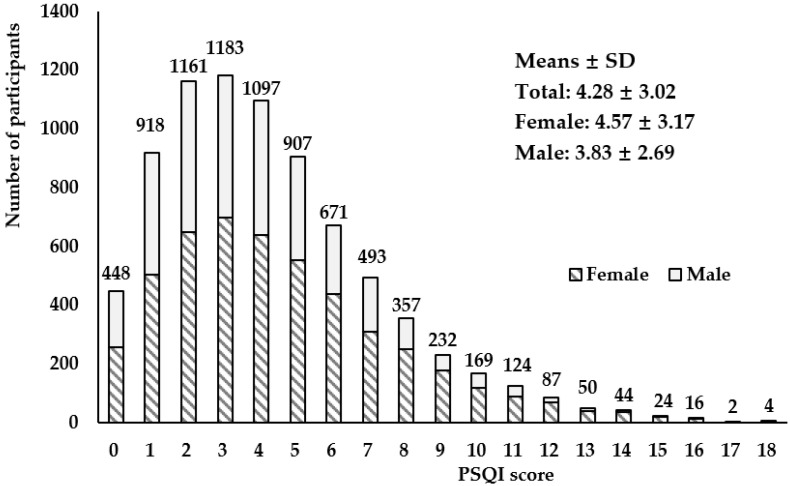
Distribution of Pittsburgh Sleep Quality Index scores among study participants (male: *n* = 3117; female: *n* = 4870).

The overall mean PSQI score of the 7987 participants was 4.28 ± 3.02, and it was 3.83 ± 2.69 in males and 4.57 ± 3.17 in females (Figure 1). The overall prevalence of poor sleep was 28.46% (PSQI score ≥ 5) (Table 2). The poor sleep distribution differences between the groups of sex, age, education level, current smoker status, current alcohol status, doing physical exercises, health related diseases of hypertension, hyperlipidemia, coronary heart disease, stroke, urarthritis, chronic bronchitis, asthma, kidney disease, cancer, schizophrenia, Parkinson’s disease and depressive disorder were significant (*p* < 0.05), while differences between the groups of BMI, marital status, being sedentary, chronic diseases of diabetes and Alzheimer’s disease were not observed. Then participant characteristics and distribution of sleep quality status are shown in Table 2.

### 3.3. Dietary Pattern Scores of the Participants

The minimum, quartiles and maximum scores of the three a priori dietary patterns (CHEI, DASH, MD) and four a posteriori dietary patterns (“Protein-rich and vegetables”, “Dairy and fruits”, “Snacks, salted food and nuts” and “Beverages”) are shown in Table 3.

The distribution differences among the study participants in the groups divided by quartiles of dietary pattern scores between good and poor sleep quality status are reported in Table 4.

The participants in higher quartiles of the three a priori dietary patterns (CHEI, DASH, MD) and one a posteriori dietary pattern (“Protein-rich and vegetables”) showed higher proportion with good sleep quality, While there were no obvious association between THE quartiles for the a posteriori “Dairy and fruits”, “Snacks, salted food and nuts” and “Beverages” dietary pattern scores and sleep quality.

### 3.4. Associations between Dietary Pattern Scores and Sleep Quality

The results of the logistic regression analysis examining the association between the dietary patterns (including the a priori and a posteriori dietary patterns) and sleep quality are presented in Table 5.

The first model of the ordinal multiple logistic regression was unadjusted, the second model was adjusted for sex and age (continuous) and the third model was further adjusted for education level (categorical), physical exercises (yes vs. no), current smoker (yes vs. no), current alcohol user (yes vs. no), hyperlipidemia (yes vs. no), hypertension (yes vs. no), coronary heart disease (yes vs. no), stroke (yes vs. no), chronic bronchitis (yes vs. no), asthma (yes vs. no), kidney disease (yes vs. no), cancer (yes vs. no), schizophrenia (yes vs. no), Parkinson’s disease (yes vs. no), and depressive disorder (yes vs. no).

For the a priori dietary patterns, significant associations between the three dietary pattern scores and poor sleep were observed. Higher CHEI (OR_Q4 vs. Q1_ = 0.811, 95% CI: 0.696–0.945, *p* < 0.01), DASH (OR_Q4 vs. Q1_ = 0.704, 95% CI: 0.603–0.823, *p* < 0.001) or MD (OR_Q4 vs. Q1_ = 0.747, 95% CI: 0.643–0.867, *p* < 0.001) scores were associated with a lower risk of poor sleep in the multivariable adjusted models (Table 5). For the a posteriori dietary patterns, the “Beverages” score (OR_Q4 vs. Q1_ = 1.180, 95% CI: 1.020–1.266, *p* < 0.05) was positively associated with poor sleep risk.

## 4. Discussion

To the best of our knowledge, this is the first study looking into the relationship between the CHEI, DASH, and MD scores and the prevalence of poor sleep in a Chinese population. The results showed that higher CHEI, DASH and MD scores were related with a lower poor sleep prevalence in the participants.

Sleep quality is an important clinical construct since it is increasingly common for people to complain about, which can be assessed using both objective and subjective methods [37]. Since the PSQI is the most widely used subjective sleep quality measure in both clinical and non-clinical populations and has good internal reliability, it can be regarded as the gold standard or established reference for self-perceived sleep quality [38]. The PSQI score of ≥5 indicated that the overall prevalence of poor sleep quality was 28.46% in this cross-sectional investigation. Although higher overall prevalence (29.57~67.30%) has been reported elsewhere in China previously [39,40,41,42], the high prevalence of about one-third in this study indicated the urgent need for preventative intervention to improve sleep quality among individuals in suburban Shanghai.

We discovered that the poor sleepers were more likely to be female and older adults, which was consistent with previous studies in different cities in China [29,30]. The emergence of this phenomenon may be due to physiological factors. With increasing age, the melatonin level decreases, which leads to a disturbance in the circadian sleep rhythm [9]. Women being more prone to poor sleep than men may be connected to changes in estrogen and female personality factors (women are more sensitive to negative emotional messages) [43]. A low education level, which often reflects low socioeconomical support factors, together with a number of lifestyle factors and chronic diseases, was also closely related to the prevalence of poor sleep.

The rising frequency of poor sleep may be partly attributed to changes in modern society’s lifestyle, where the dietary pattern may be a significant and independent influence affecting sleep quality. Using both a priori and a posteriori dietary patterns, we first looked into the relationships between dietary patterns and sleep quality in a Shanghai, China, suburban population in this cross-sectional study. After being adjusted for those covariates that may affect sleep quality, our findings showed that the three a priori dietary pattern scores of the CHEI, DASH and MD and one a posteriori dietary pattern “Beverages” were associated with sleep quality in the study population.

The CHEI, calculated by higher intakes of 12 adequacy components (including total grains, whole grains and mixed beans, tubers, total vegetables, dark vegetables, fruits, dairy, soybeans, fish and seafood, poultry, eggs, seeds and nuts) and lower intakes of 5 limitation components (including red meat, cooking oils, sodium, added sugars, and alcohol), is a tool that has been shown to have strong validity and reliability for assessing the overall quality of a meal in compliance with the most recent Dietary Guidelines for Chinese (DGC-2016) [44]. Rich in fruits, vegetables, and low-fat dairy products, the DASH diet is acknowledged as an effective dietary strategy to lower blood pressure (BP) in persons with and without hypertension [45]. Over the course of several decades, the definition of the MD has changed and evolved. Originally, it was defined as being low in saturated fat and high in vegetable oils in Greece and Southern Italy. Today, the score is evaluated based on the high consumption of fruit, vegetables, fish, whole grains, and olive oil, as well as the limited consumption of red and processed meat and alcohol [16]. One feature that all three indices have in common is that the higher the score, the more whole grains, fruits, vegetables, and nuts one consumes, and the lower one consumes red meat. The similarities between these indicators could aid in identifying the essential food elements for more studies examining the connection between nutrition and sleep.

The MD or DASH dietary pattern has been shown in earlier research to correlate increased intakes of fruits, vegetables, and legumes with better sleep characteristics [46,47,48]. On the other hand, frequent use of sugar-rich beverages and a high red meat intake has been linked to detrimental effects on both the quantity and quality of sleep [49]. The foods and nutrients and their potential combinations in the particular dietary pattern may contribute to the biological plausibility of this phenomenon. For instance, diet may influence sleep via melatonin and its biosynthesis from tryptophan [50]. Better sleep patterns have been linked to an increase in tryptophan availability and brain uptake following carbohydrate eating, particularly from fruits, vegetables, and whole grains. Tryptophan is a precursor of serotonin and melatonin [51,52]. Moreover, consuming foods containing tryptophan or melatonin, including milk, tomatoes, kiwis, cherries and melatonin-containing meals, has been linked to improved sleep habits [53]. It has been demonstrated that providing particular foods high in melatonin or tryptophan can enhance the quality of sleep, and whole diets high in fruits, vegetables, legumes, and other dietary sources of melatonin and tryptophan predict better sleep quality [50].

The current study identified four a posteriori dietary patterns, and significant correlation was found only between the scores for the dietary pattern “Beverages” and the prevalence of poor sleep. The correlation showed that the dietary pattern with higher consumption of beverages (including sugar-sweetened beverages and juice) was associated with a higher poor sleep prevalence in the study population.

In this study, the a posteriori dietary pattern “Beverages” was derived from the food group items of “sugar-sweetened beverages” and “juice” on the food frequency questionnaire. Natural fruit juices have a sugar composition quite similar to that of sugar-sweetened beverages. Fruit juices may include lower average concentrations of certain beneficial elements, such as fiber, polyphenols, and other phytochemicals, which are largely lost during the juicing process, in addition to having comparatively greater simple sugar contents as compared to whole fruits [54]. The association between beverage intake and poor sleep quality may relate to the high fructose content in liquid form, providing only “empty” calories with little nutritional value, which may increase the glucose level and influence the normal circadian rhythm system that regulates the normal metabolic process of the body, and so lead to poor sleep [55].

The strengths of this study are the inclusion of a relatively large number of representative Shanghai suburb native adult residents based on a large population-based cohort, and the utilization of dietary patterns that allowed the investigation of synergistic effects of nutrients.

We must, however, also be aware of the limitations of our research. Firstly, because the a posteriori dietary pattern technique relies on sample information, the pattern that is generated is quite particular to the diet of the group under study. It is possible that the patterns or conclusions from this study cannot be applied to other target groups. Secondly, in spite of the questionnaires utilized to investigate the suggested research topic being well-established tools, the use of self-reported FFQs and PSQI questionnaires may be influenced by biases related to recall and social desirability, leading to the under-reporting phenomenon. Thirdly, even with our extensive covariate adjustment, residual and unmeasured confounding cannot be totally eliminated. Fourthly, the observed associations in this study cannot be interpreted as causative due to the cross-sectional methodology. Furthermore, our study is unable to establish temporal correlations between the exposure and outcome variables because the dietary habits and sleep quality were examined at the same time. It is anticipated that future research employing intervention trials and longitudinal data will clarify or validate this causal relationship.

## 5. Conclusions

In summary, a better diet quality, as measured by higher CHEI, DASH and MD scores, as well as lower consumption of beverages, was associated with better sleep quality in Shanghai suburban adults. To validate this relationship and find out if relevant dietary changes can lessen poor sleep in Chinese adults, more prospective research is required.

## Figures and Tables

**Table 1:** Factor loadings to determine the association between food groups and factors representing dietary patterns ^a^.

Food Groups	Dietary Patterns ^b^			
	Factor 1: “Protein-Rich and Vegetables”	Factor 2: “Dairy and Fruits”	Factor 3: “Snacks, Salted Food and Nuts”	Factor 4: “Beverages”
Meat	**0.707**	−0.086	0.108	0.115
Fish and seafood	**0.676**	0.105	0.135	−0.056
Poultry	**0.659**	−0.064	0.087	0.156
Vegetables	**0.572**	0.104	−0.076	−0.102
Soybeans and soy products	**0.457**	0.206	0.117	0.233
Dairy and dairy products	0.153	**0.664**	0.064	0.116
Fruits	0.282	**0.549**	−0.035	0.052
Snacks	0.007	0.059	**0.650**	0.109
Salted food	−0.036	−0.305	**0.590**	0.094
Nuts and seeds	0.169	0.160	**0.460**	−0.111
Sugar-sweetened beverages	0.063	−0.054	0.056	**0.777**
Juice	0.050	0.278	−0.027	**0.664**
Eggs	0.306	0.054	0.370	−0.013
Grain and tubers	0.303	−0.440	0.025	−0.122
Whole grain and mixed beans	0.290	0.241	0.256	−0.174
Alcohol	0.259	−0.359	−0.270	0.111

^a^ The factor loading scores and correlation coefficients are the same when promax rotation is used. Bold text indicates factor loadings with absolute values greater than 0.40. The only loading that is bolded for food groups that load more than one dietary pattern is the highest absolute loading value. ^b^ The following dietary patterns explain the variation: 16.776% for “Protein-rich and vegetables”, 8.769% for “Dairy and fruits”, 7.375% for “Snacks, salted food, and nuts”, and 7.00% for “Beverages”.

**Table 2:** Characteristics of the participants by sleep quality (*n* = 7987).

Characteristics		Total	Good Sleeper (PQIS < 5)	Poor Sleeper (PQIS ≥ 5)	*p* Value ^a^
Age (years)		53.19 ± 13.47	52.55 ± 13.85	54.79 ± 12.32	<0.001
BMI (kg/m^2^)		23.68 ± 4.91	23.70 ± 4.95	23.64 ± 4.90	0.615
Energy intake (kcal/day)		1745.35 ± 683.70	1757.95 ± 685.15	1713.67 ± 679.15	0.009
Total, N (%)		7987 (100)	5714 (71.54)	2273 (28.46)	
Sex					
Male		3117 (39.03)	2417 (77.54)	700 (22.46)	<0.001
Female		4870 (60.97)	3297 (67.70)	1573 (32.30)	
Age					
<40 years		1610 (20.16)	1283 (79.69)	327 (20.31)	<0.001
40–60 years		3910 (48.95)	2682 (68.59)	1228 (31.41)	
60–74 years		2467 (30.89)	1749 (70.90)	718 (29.10)	
BMI					
Underweight		467 (5.85)	322 (68.95)	145 (31.05)	0.592
Normal		3615 (45.26)	2602 (71.98)	1013 (28.02)	
Overweight		2853 (35.72)	2037 (71.40)	816 (28.60)	
Obese		1052 (13.17)	753 (71.58)	299 (28.42)	
Education					
Primary school or below		2726 (34.13)	1845 (67.68)	881 (32.32)	<0.001
Secondary school or above		5261 (65.87)	3869(73.54)	1392 (26.46)	
Marital status					
Married		7384 (92.45)	5300 (71.78)	2084 (28.22)	0.103
Other ^b^		603 (7.55)	414 (68.66)	189 (31.34)	
Lifestyle variables					
Current smoker ^c^	Yes	1669 (20.90)	1258 (75.37)	411 (24.63)	<0.001
	No	6318 (79.10)	4456 (70.53)	1862 (29.47)	
Current alcohol user ^d^	Yes	787 (9.85)	490 (62.26)	297 (37.74)	<0.001
	No	7200 (90.15)	5224 (72.56)	1976 (27.44)	
Physical exercise ^e^	Yes	2304 (28.85)	1715 (74.44)	589 (25.56)	<0.001
	No	5683 (71.15)	3999 (70.37)	1684 (29.63)	
Being sedentary > 6 h/day	Yes	1877 (23.50)	1359 (72.40)	518 (27.60)	0.344
	No	6110 (76.50)	4355 (71.28)	1755 (28.72)	
Related diseases					
Hypertension	Yes	2424 (30.35)	1632 (67.33)	792 (32.67)	<0.001
	No	5563 (69.65)	4082 (73.38)	1481 (26.62)	
Hyperlipidemia	Yes	944 (11.82)	724 (76.69)	220 (23.31)	<0.001
	No	7043 (99.18)	4990 (70.85)	2053 (29.15)	
Diabetes	Yes	575 (7.20)	393 (68.35)	182 (31.65)	0.078
	No	7412 (92.80)	5321 (71.79)	2091 (28.21)	
Coronary heart disease	Yes	430 (5.38)	238 (55.35)	192 (44.65)	<0.001
	No	7557 (94.62)	5476 (72.46)	2081 (27.54)	
Stroke	Yes	142 (1.78)	86 (60.56)	56 (39.44)	0.003
	No	7845 (98.22)	5628 (71.74)	2217 (28.26)	
Urarthritis	Yes	413 (5.17)	235 (56.90)	178 (43.10)	<0.001
	No	7574 (94.83)	372 (48.75)	362 (47.44)	
Chronic bronchitis	Yes	513 (6.42)	311 (60.62)	202 (39.38)	<0.001
	No	7474 (93.58)	5403 (72.29)	2071 (27.71)	
Asthma	Yes	166 (2.08)	94 (56.63)	72 (43.37)	<0.001
	No	7821 (97.92)	5620 (71.86)	2201 (28.14)	
Kidney disease	Yes	981 (12.28)	592 (60.35)	389 (39.65)	<0.001
	No	7006 (87.72)	5122 (73.11)	1884 (26.89)	
Cancer	Yes	57 (0.71)	31 (54.39)	26 (45.61)	<0.001
	No	7930 (99.29)	5683 (71.66)	2247 (28.34)	
Schizophrenia	Yes	22 (0.28)	11 (50.00)	11 (50.00)	0.025
	No	7965 (99.72)	5703 (71.60)	2262 (28.40)	
Parkinson’s disease	Yes	46 (0.58)	22 (47.83)	24 (52.17)	<0.001
	No	7941 (99.42)	5692 (71.68)	2249 (28.32)	
Alzheimer’s disease	Yes	20 (0.25)	13 (65.00)	7 (35.00)	0.516
	No	7967 (99.75)	5701 (71.56)	2266 (28.44)	
Depressive disorder	Yes	31 (0.39)	10 (32.26)	21 (67.74)	<0.001
	No	7956 (99.61)	5704 (71.69)	2252 (28.31)	

^a^ Continuous variables are presented as mean ± SD, categorical variables are presented as frequency (percentages), *p*-value between participants in the good sleep quality and poor sleep groups by Student’s independent *t* test or chi square test; ^b^ Widowed, separated, divorced, single or never married; ^c^ Smoking at least once a day for at least six months; ^d^ Drinking alcohol at least three times a week for at least six months; ^e^ Performing physical activity lasting at least 10 min per week.

**Table 3:** Dietary pattern scores of the participants (*n* = 7987).

Dietary Pattern Score	Minimum	Quartiles			Maximum
		25th Percentile	Median	75th Percentile	
CHEI	20.85	49.7	57.33	64.85	84.09
DASH	9.50	21.50	24.50	27.50	37.50
MDS	0.00	4.00	5.00	6.00	9.00
Factor 1: Protein-rich and vegetables	−2.71	−0.71	−0.22	0.5	7.62
Factor 2: Dairy and fruits	−8.84	−0.59	−0.1	0.51	7.97
Factor 3: Snacks, salted food and nuts	−8.02	−0.64	−0.23	0.39	10.86
Factor 4: Beverages	−3.71	−0.45	−0.23	0.08	13.20

**Table 4:** Overall sleep quality status distribution of the study participants by quartiles of dietary pattern scores.

Dietary Pattern Score	Q1	Q2	Q3	Q4	*p* Value ^a^
CHEI, Medians	44.56	53.52	61.01	69.53	
Good Sleep Quality (PQIS < 5), *n* (%)	1339 (67.0)	1423 (71.3)	1456 (72.9)	1496 (74.9)	
Poor Sleep Quality (PQIS ≥ 5), *n* (%)	659 (33.0)	572 (28.7)	542 (27.1)	500 (25.1)	<0.001
DASH, Medians	20.50	23.50	26.50	29.50	
Good Sleep Quality (PQIS < 5), *n* (%)	1534 (68.6)	1579 (71.2)	1462 (73.2)	1139 (74.2)	
Poor Sleep Quality (PQIS ≥ 5), *n* (%)	702 (31.4)	640 (28.8)	534 (26.8)	397 (25.8)	<0.001
MDS, Medians	3.00	5.00	6.00	7.00	
Good Sleep Quality (PQIS < 5), *n* (%)	2406 (68.1)	1146 (72.7)	1128 (75.2)	1034 (75.0)	
Poor Sleep Quality (PQIS ≥ 5), *n* (%)	1125 (31.9)	431 (27.3)	372 (24.8)	345 (25.0)	<0.001
Factor 1: Protein-rich and vegetables, Medians	−0.97	−0.47	0.08	1.12	
Good Sleep Quality (PQIS < 5), *n* (%)	1368 (68.5)	1417 (70.9)	1458 (73.0)	1471 (73.7)	
Poor Sleep Quality (PQIS ≥ 5), *n* (%)	628 (31.5)	581 (29.1)	538 (27.0)	526 (26.3)	<0.001
Factor 2: Dairy and fruits, Medians	−0.96	−0.33	0.17	1.08	
Good Sleep Quality (PQIS < 5), *n* (%)	1440 (72.1)	1365 (68.4)	1455 (72.8)	1454 (72.8)	
Poor Sleep Quality (PQIS ≥ 5), *n* (%)	556 (27.9)	632 (31.6)	543 (27.2)	542 (27.2)	0.003
Factor 3: Snacks, salted food and nuts, Medians	−0.85	−0.44	0.04	1.32	
Good Sleep Quality (PQIS < 5), *n* (%)	1442 (72.2)	1417 (70.9)	1417 (71.0)	1438 (72.0)	
Poor Sleep Quality (PQIS ≥ 5), *n* (%)	554 (27.8)	581 (29.1)	579 (29.0)	559 (28.0)	0.712
Factor 4: Beverages, Medians	−0.63	−0.33	−0.11	1.16	
Good Sleep Quality (PQIS < 5), *n* (%)	1452 (72.7)	1405 (70.4)	1409 (70.6)	1448 (72.5)	
Poor Sleep Quality (PQIS ≥ 5), *n* (%)	545 (27.3)	592 (29.6)	588 (29.4)	548 (27.5)	0.199

^a^ The distribution differences of the quartiles groups between the good and poor sleepers tested by the chi-square test.

**Table 5:** Associations between each dietary pattern score and sleep quality.

Dietary Pattern Score	Model 1 ^a^			Model 2 ^b^			Model 3 ^c^		
	OR	95% CI	*p* Value	OR	95% CI	*p* Value	OR	95% CI	*p* Value
CHEI									
Q1	Reference			Reference			Reference		
Q2	0.817	0.714–0.934	0.003	0.896	0.781–1.029	0.121	0.933	0.810–1.074	0.331
Q3	0.756	0.660–0.866	<0.001	0.83	0.720–0.956	0.010	0.875	0.757–1.011	0.069
Q4	0.679	0.592–0.779	<0.001	0.742	0.640–0.860	<0.001	0.811	0.696–0.945	0.007
DASH									
Q1	Reference			Reference			Reference		
Q2	0.886	0.779–1.007	0.063	0.817	0.717–0.932	0.003	0.833	0.729–0.952	0.007
Q3	0.798	0.698–0.912	0.001	0.721	0.628–0.828	<0.001	0.758	0.659–0.873	<0.001
Q4	0.762	0.659–0.880	<0.001	0.667	0.574–0.776	<0.001	0.704	0.603–0.823	<0.001
MDS									
Q1	Reference			Reference			Reference		
Q2	0.804	0.705–0.917	0.001	0.796	0.695–0.910	0.001	0.819	0.714–0.938	0.004
Q3	0.705	0.615–0.809	<0.001	0.710	0.616–0.818	<0.001	0.744	0.644–0.859	<0.001
Q4	0.714	0.620–0.822	<0.001	0.705	0.609–0.817	<0.001	0.747	0.643–0.867	<0.001
Factor 1: Protein-rich and vegetables									
Q1	Reference			Reference			Reference		
Q2	0.893	0.780–1.022	0.101	1.012	0.881–1.163	0.865	1.073	0.931–1.236	0.329
Q3	0.804	0.701–0.922	0.002	0.947	0.822–1.091	0.447	1.014	0.877–1.172	0.854
Q4	0.779	0.679–0.894	<0.001	1.000	0.864–1.158	0.999	1.061	0.913–1.232	0.44
Factor 2: Dairy and fruits									
Q1	Reference			Reference			Reference		
Q2	1.036	0.901–1.190	0.62	1.072	0.932–1.233	0.332	1.048	0.907–1.210	0.527
Q3	1.242	1.084–1.424	0.002	0.859	0.741–0.996	0.045	0.875	0.752–1.019	0.086
Q4	1.001	0.871–1.151	0.987	0.868	0.745–1.011	0.069	0.913	0.780–1.069	0.259
Factor 3: Snacks, salted food and nuts									
Q1	Reference			Reference			Reference		
Q2	1.067	0.930–1.225	0.354	1.056	0.919–1.213	0.446	1.071	0.930–1.233	0.342
Q3	1.064	0.927–1.221	0.38	1.040	0.905–1.195	0.584	1.06	0.921–1.222	0.416
Q4	1.012	0.881–1.162	0.868	0.936	0.813–1.077	0.354	0.972	0.843–1.121	0.696
Factor 4: Beverages									
Q1	Reference			Reference			Reference		
Q2	1.123	0.978–1.288	0.099	1.097	0.955–1.261	0.189	1.083	0.940–1.247	0.27
Q3	1.112	0.969–1.276	0.131	1.048	0.912–1.205	0.508	1.046	0.907–1.206	0.538
Q4	1.008	0.877–1.159	0.907	1.165	1.010–1.345	0.037	1.180	1.020–1.266	0.026

Note: ^a^ Model 1 unadjusted model. ^b^ Model 2 adjusted for sex and age. ^c^ Model 3 adjusted for sex, age, education level (categorical), physical exercises (yes vs. no), current smoker (yes vs. no), current alcohol user (yes vs. no), hyperlipidemia (yes vs. no), hypertension (yes vs. no), coronary heart disease (yes vs. no), stroke (yes vs. no), chronic bronchitis (yes vs. no), asthma (yes vs. no), kidney disease (yes vs. no), cancer (yes vs. no), schizophrenia (yes vs. no), Parkinson’s disease (yes vs. no), and depressive disorder (yes vs. no).

## Data Availability

The data presented in this study are available on request from the corresponding author due to privacy of the study participants. (specify the reason for the restriction, e.g., privacy, legal or ethical reasons).

## Supplementary Materials

The following supporting information can be downloaded at https://www.mdpi.com/article/10.3390/nu16081165/s1. Table S1: Components and scoring of the CHEI, DASH and MD; Table S2: Food groups and items in the food frequency questionnaire.
