# Supplementary material for: Healthier Dietary Patterns Are Associated with Better Sleep Quality among Shanghai Suburban Adults: A Cross-Sectional Study
Source: Nutrients. 2024 Apr 13;16(8):1165. doi: 10.3390/nu16081165 (PMC11054136; doi:10.3390/nu16081165)
Supplement: Supplementary file 1 [file nutrients-16-01165-s001.zip › nutrients-2884148-supplementary.pdf]

Supplemental table S1. Components and scoring of the CHEI, DASH and MD

Component	Intake of minimum score	Intake of maximum score	Score range
CHEI-2016			0 ~ 100
1. Total Grains (sp)	0	≥ 2.5	0 ~ 5
2. Whole Grains and Mixed Beans (sp)	0	≥ 0.6	0 ~ 5
3. Tubers (sp)	0	≥ 0.3	0 ~ 5
4. Total Vegetables (sp)	0	≥ 1.9	0 ~ 5
5. Dark Vegetables (sp)	0	≥ 0.9	0 ~ 5
6. Fruits (sp)	0	≥ 1.1	0 ~ 10
7. Dairy (sp)	0	≥ 0.5	0 ~ 5
8. Soybeans (sp)	0	≥ 0.4	0 ~ 5
9. Nuts (sp)	0	≥ 0.4	0 ~ 5
10. Fish and Seafood (sp)	0	≥ 0.6	0 ~ 5
11. Poultry (sp)	0	≥ 0.3	0 ~ 5
12. Eggs (sp)	0	≥ 0.5	0 ~ 5
13. Red Meat (sp)	≥ 3.5	≤ 0.4	0 ~ 5
14. Cooking Oils (g)	≥ 32.6	≤ 15.6	0 ~ 10
15. Sodium (mg)	≥ 3608	≤ 1000	0 ~ 10
16. Added Sugars (% of Energy Intake)	≥ 20%	≤ 10%	0 ~ 5
17. Alcohol (g)	≥ 60 (male); ≥40(Female)	≤ 25(male); ≤15(Female)	0 ~ 5
DASH [a]			8 ~ 40
1. Fruits (sp)	Quintile 1	Quintile 5	1 ~ 5
2. Vegetables (sp)	Quintile 1	Quintile 5	1 ~ 5
3. Nuts and legumes (sp)	Quintile 1	Quintile 5	1 ~ 5
4. Low-fat dairy (sp)	Quintile 1	Quintile 5	1 ~ 5
5. Whole grains (sp)	Quintile 1	Quintile 5	1 ~ 5
6. Red and processed meats (sp)	Quintile 5	Quintile 1	1 ~ 5
7. Sugar-sweetened beverages (g)	Quintile 5	Quintile 1	1 ~ 5
8. Sodium (mg)	Quintile 5	Quintile 1	1 ~ 5
MD			0 ~ 9
1. Vegetables (sp)	Below the median	Above the median	0 ~ 1
2. Fruits (sp)	Below the median	Above the median	0 ~ 1
3. Nuts (sp)	Below the median	Above the median	0 ~ 1
4. Whole grains (sp)	Below the median	Above the median	0 ~ 1
5. Legumes (sp)	Below the median	Above the median	0 ~ 1
6. Fish and Seafood (sp)	Below the median	Above the median	0 ~ 1
7. Monounsaturated/saturated fat ratio	Below the median	Above the median	0 ~ 1
8. Red and processed meats (sp)	Below the median	Above the median	0 ~ 1
9. Alcohol (g)	<5, > 15	5 ~ 15	0 ~ 1

CHEI: Chinese Healthy Eating Index; DASH: Dietary Approaches to Stop Hypertension; MD: Mediterranean Diet; SP: Standard Portion; [a]: Intakes between the minimum and maximum levels are scored proportionately.

Supplemental table S2. Food groups and items in Food frequency questionnaire

Food groups	Items in Food frequency questionnaire
Grains and tubers	Rice and rice products, wheat and wheat products, potato, cassava, yam, fried dough foods and potato chips, etc.
Whole Grains and Mixed Beans	Sorghum, millet, oats, mung beans and red beans, etc.
Soybeans and soy products	Soybean, soy milk, tofu, etc.
Vegetables	Dark-green leafy vegetables, red and yellow vegetables, stem, melon, root, algae and mushroom, etc.
Dairy and dairy products	Fresh milk, yogurt, powdered milk and cheese, etc.
Fruits	Fresh and canned (no added sugar) fruits, etc.
Nuts and Seeds	Peanuts, walnuts, almonds and melon seeds, etc.
Meats	Pork, beef and lamb, animal viscera and processed meat, etc.
Poultry	Chicken, duck and goose, etc.
Eggs	Whole eggs, yolk, white, preserved eggs, etc.
Fish and seafood	Freshwater fish, sea fish, shrimp, crab, shellfish, etc.
Alcohol	Chinese rice wine, beer, wine, white wine and whiskey, etc.
Sugar-sweetened beverages	Carbonated drinks, flavored drinks, soft drinks, etc.
Juice	Pure fruit juice.
Snacks	Sugar, jam, honey, cakes, chocolate, mooncake, potato chips, ice cream, etc.
Salted food	Preserved meat, preserved vegetables, sausages, etc.
